# The herpes simplex virus 1 Us3 kinase is involved in assembly of membranes needed for viral envelopment and in distribution of glycoprotein K

**DOI:** 10.12688/f1000research.19194.1

**Published:** 2019-05-23

**Authors:** Kurt Tobler, Claudia Senn, Elisabeth M. Schraner, Mathias Ackermann, Cornel Fraefel, Peter Wild

**Affiliations:** 1 Institute of Virology, University of Zürich, Zürich, CH-8057, Switzerland

**Keywords:** HSV-1, envelopment, nuclear membranes, Golgi membranes, gK, immunogoldlabeling, immunofluorescence, TEM

## Abstract

**Background** **:** Capsids of herpes simplex virus 1 (HSV-1) are assembled in cell nuclei, released into the perinuclear space by budding at the inner nuclear membrane acquiring tegument and envelope. Alternatively, capsids gain access to the cytoplasm via dilated nuclear pores. They are enveloped by Golgi membranes. Us3 is a non-essential viral kinase that is involved in nucleus-to-cytoplasm translocation, preventing apoptosis and regulation of phospholipid-biosynthesis. Us3-deletion mutants **(**HSV-1∆Us3) accumulate in the perinuclear space. Nuclear and Golgi membranes proliferate, and homogeneous, proteinaceous structures of unknown identity are deposited in nuclei and cytoplasm. Glycoprotein K (gK), a highly hydrophobic viral protein, is essential for production of infectious progeny virus but, according to the literature, exclusively vital for envelopment of capsids by Golgi membranes. In the absence of Us3, virions remain stuck in the perinuclear space but mature to infectivity without reaching Golgi membranes, suggesting further function of gK than assumed.

**Methods** **:** We constructed a HSV-1∆Us3 mutant designated CK177∆Us3gK-HA, in which gK was hemagglutinin (HA) epitope-tagged in order to localize gK by immunolabeling using antibodies against HA for light and electron microscopy.

**Results** **:** CK177∆Us3gK-HA-infected Vero cells showed similar alterations as those reported for other HSV-1∆Us3, including accumulation of virions in the perinuclear space, overproduction of nuclear and Golgi membranes containing electron dense material with staining property of proteins. Immunolabeling using antibodies against HA revealed that gK is overproduced and localized at nuclear membranes, perinuclear virions stuck in the perinuclear space, Golgi membranes and on protein deposits in cytoplasm and nuclei.

**Conclusions** **:** Us3 is involved in proper assembly of membranes needed for envelopment and incorporation of gK. Without Us3, virions derived by budding at nuclear membranes remain stuck in the perinuclear space but incorporate gK into their envelope to gain infectivity.

## Introduction

Capsids of herpes simplex virus 1 (HSV-1) are assembled in host cell nuclei, transported to the nuclear periphery and translocated to the perinuclear space (PNS) or into the cytosol ( Roizman *et al.*, 2007). The HSV-1 Us3 kinase phosphorylates numerous viral and cellular substrates ( Kato & Kawaguchi, 2018). It is involved in nucleus-to-cytoplasm capsid translocation ( Kato *et al.*, 2009; Mou *et al.*, 2009; Reynolds *et al.*, 2002; Ryckman & Roller, 2004; Wisner *et al.*, 2009), in regulation of phospholipid synthesis induced by HSV-1 ( Sutter *et al.*, 2012; Wild *et al.*, 2012), and in blocking apoptosis induced by HSV-1 ( Galvan & Roizman, 1998; Goodkin *et al.*, 2004; Leopardi *et al.*, 1997). The Golgi complex plays a significant role in apoptosis ( Cheng *et al.*, 2010; Hicks & Machamer, 2005), in membrane biosynthesis together with the endoplasmic reticulum ( Smirle *et al.*, 2013), and in HSV-1 envelopment ( Roizman *et al.*, 2014). Glycoprotein K (gK) is a hydrophobic transmembrane protein of the viral envelope ( Mo & Holland, 1997) encoded by the UL53 gene ( Bond & Person, 1984; Hutchinson *et al.*, 1992). gK is essential for efficient envelopment by Golgi membranes ( Baines *et al.*, 1991; Melancon *et al.*, 2007) and for production of infectious progeny virus ( Chouljenko *et al.*, 2012). HSV-1ΔUs3 virions accumulate in the PNS. Nuclear and Golgi membranes become severely altered by insertion of proteins of unknown nature ( Wild *et al.*, 2015). The essentiality of gK in envelopment by Golgi membranes prompted us to identify gK in cells infected with HSV-1ΔUs3. Because of the hydrophobicity of gK, we constructed a HSV-1ΔUs3 with a hemagglutinin (HA) tag at gK, CK177ΔUs3gK-HA. Immunogold labeling shows that gK localizes on amorphous protein structures, nuclear and Golgi membranes, and, importantly, on virions in the PNS.

## Methods

### Cells and viruses

Vero cells (European Collection of Cell Cultures, ECACC, 84113001) were grown in Dulbecco’s modified minimal essential medium (DMEM; 31885-023; Gibco, Bethesda, MD, USA) supplemented with penicillin (100 U/ml), streptomycin (100 μg/ml) (Anti-Anti, 15240-062, Gibco) and 10% fetal bovine serum (FBS; 2-01F10-I, Bio Concept, Allschwil, Switzerland). The Us3-deletion mutant R7041ΔUs3 was kindly provided by Bernard Roizman (The Marjorie B. Kovler Viral Oncology Laboratories, University of Chicago, Illinois, USA). Wild-type (wtHSV-1) strain F (Ejercito *et al.*, J. Gen. Virol. 2:357–364, 1968), R7041ΔUs3 were propagated in Vero cells.

### Generation of recombinants CK177ΔUs3gK-HA and CK177gK-HA

Recombineering of pYEbac102 ( Tanaka *et al.*, 2003) was done in two steps in *Escherichia coli* strain SW102 ( Warming *et al.*, 2005) First, a galK expression cassette was amplified by PCR (Phusion R, High-Fidelity DNA Polymerase; M0530L, BioLabs. Ipswich, MA, USA, according to manufacturer’s recommendations) with homology arms flanking UL53 using the following primers: primer forward, 5’- tct tcg gtg cca gtc cgc tgc acc gat gta ttt acg cgg tac gcc cca ccg CCT GTT GAC AAT TAA TCA TCG GCA -3’; primer reverse, 5’- gtt tcc aat ttg cat atg ccg tta cgg ttt ccg ccg gcc tgg atg tga cgt TCA GCA CTG TCC TGC TCC TT -3’ (binding sequences in capitals). This amplimer was DpnI treated to remove template DNA, purified, and electroporated into competent and induced *E. coli* strain SW102 carrying pYEbac102 ( Tanaka *et al.*, 2003). Recombinant colonies were selected for growth on galactose plates. The BAC DNA carrying the HSV genomic sequence with deleted UL53 was designated pYEbac102ΔUL53. Second, a kanamycin resistant cassette was amplified by PCR on pBSrspL (Genes Bridges, Dresden, Germany; kanamycin resistant cassette) with the following primers for Us3rpsL: primer forward, 5’-ctt ccc aca cca cac cac cca gcg agg ccg agc gcc tgt gtc atc tgc aGG GCC TGG TGA TGA TGG CGG GAT CG-3’, primer reverse 5’-aga tca cca gac cgg cgc tcc aaa tgt cga cgg tcg tgg tat acg gat ccT CAG AAG AAC TCG TCA AGA AGG-3’ (binding sequences in capitals). The primers were chosen to yield the same deletion of Us3 as described by Purves *et al.* (1987). This amplimer was DpnI-treated, purified, and electroporated into competent and induced *E. coli* strain SW102 carrying pYEbac102ΔUL53. Recombinant colonies were selected for growth on kanamycin plates. Finally, fHSVgKgalKΔUS3, pCS177 (HA tagged gK and flanking sequences, see below) and pCMV-Cre (Cre recombinase under CMV promoter) DNA was mixed and co-transfected into Vero cells using Lipofectamine 2000 (11668027, Thermo Fisher Scientific, Rockford, IL, USA) according to the manufacturer’s recommendations. Rescued progeny virus with deleted Us3 and BAC backbone but HA-tagged gK, designated CK177ΔUs3gK-HA, was propagated in Vero cells.

Plasmid pCS177 containing the UL53 gene with a carboxy terminal HA tag and flanking sequence to UL53 that extend 0.3 kbp upstream and 0.4 kbp downstream of the UL53 gene was constructed as follows. First, downstream sequence of UL53 was amplified by PCR from wtHSV-1 genome (primer forward, 5’- gat ctc tag acg tca cat cca ggc cgg cgg aa - 3’; primer reverse, 5’- gat cga gct cag gCC TCC GGC ACA GAC AAG GAC CAA T -3’; HSV-1 sequences in capitals). The resulting PCR product was digested with SacI and XbaI and cloned into the SacI and XbaI sites in pBluescript II KS(+), resulting in plasmid pCS176. Second, the UL53 gene with its upstream flanking sequence was amplified by PCR from wtHSV-1 using a reverse primer containing nucleotides of HA tag (primer forward, 5’- gat caa gct tag gcc tgg gtc ggt aca acg tac agc cgg at - 3’; primer reverse, 5’- gat ctc tag aTC Acc atg gag cat aat ctg gaa cat cat atg gat aTA CAT CAA ACA GGC GCC TCT gga -3’; HSV-1 sequences in capitals). Following PCR, the DNA product was digested with HindIII and XbaI and inserted into these sites in pCS176, resulting in plasmid pCS177. The StuI fragment of pCS177 containing UL53-HA gene with flanking sequence was co-transfected together with pYEbac102ΔUL53 and pCMV-Cre into Vero cells. gK-HA expression was identified using indirect immunofluorescence and one virus stock expressing gK-HA was designated CK177gK-HA.

### Infection of cells

Vero cells were grown for 2 days on cover slips (Assistent, Sondheim, Germany) for immunofluorescence, on sapphire disks (100.00174, Bruegger, Minusion, Switzerland) placed in 6 well plates for TEM, or in 75 cm ^2^ cell culture flasks for immunogoldlabeling, for 2 days prior to inoculation with CK177ΔUs3gK-HA, R7041ΔUs3, CK177gK-HA or wtHSV-1 at a multiplicity of infection (MOI) of 1 plaque-forming unit (pfu)/ml.

### Cryo-fixation for transmission electron microscopy

Cells on sapphire disks were frozen 16 to 24 hpi in a high-pressure freezer (HPM010; BAL-TEC Inc., Balzers, Liechtenstein) and prepared for sectioning as described in detail previously ( Wild *et al.*, 2018; Wild *et al.*, 2002). Cells were analyzed in a transmission electron microscope (CM12; FEI, Eindhoven, The Netherlands) equipped with a CCD camera (Ultrascan 1000; Gatan, Pleasanton, CA, USA).

### Immunofluorescence

Cells inoculated with CK177ΔUs3gK-HA or CK177gK-HA at MOI 1 were incubated for 20 h, briefly washed with PBS, fixed with 2% formaldehyde for 25 min at room temperature, washed with cold PBS, permeabilized with 0.1% Triton-X-100 at room temperature for 7 min, and blocked with 3% bovine serum albumin in phosphate-buffered saline (PBS) containing 0.05% Tween 20 (PBST). Cells were labeled with mouse monoclonal HA-probe antibodies (1:500) (SC-7392, Santa Cruz Biotechnology, Dallas, TX, USA), followed by anti-mouse Alexa 488-conjugated secondary antibodies (1:500) (A1101; Thermo Fisher) as well as with polyclonal antibodies (1:500) raised in rabbits against Us3 (kindly provided by Bernard Roizman) followed by anti-rabbit Alexa 594-conjugated secondary antibodies (1:500) (A11037; Thermo Fisher). After staining nuclei with 4',6-diamidino-2-phenylindol (DAPI; Roche, Mannheim, Germany), cells were embedded in glycergel mounting media (C0563; Dako North America, Carpinteria, CA, USA) and 25 mg/ml DABCO (1,4-diazabicyclo [2.2.2] octane; 33480, Fluka, Buchs, Switzerland) and analyzed using a confocal laser scanning microscope (SP2, Leica, Wetzlar, Germany).

### Immunogold labeling

Inoculated cells were harvested at 20 hpi and prepared according to ( Tokuyasu, 1973; Tokuyasu, 1980). Cells fixed with 4% formaldehyde in 0.1 M Na-phosphate, pH 7.4, for 2 h at room temperature were scraped from the flasks, washed three times with 0.1 M Na-phosphate by centrifugation at 13,000 *g* for 30 s, and pelleted in 12% gelatine by centrifugation at 13,000 *g* for 3 min at 37°C. After gelation at 4°C, 1 mm ^3^ blocks were immersed overnight in 2.3 M sucrose constantly rotating at 4°C. The infiltrated blocks were mounted on specimen holders, frozen by plunging into liquid nitrogen, and placed into the cryo-microtome (UC6, Leica, Vienna, Austria) at -120°C. Ultra-thin sections of 80–100 nm were collected on carbon-coated formvar films mounted on hexagonal 100 mesh/inch copper grids. Sections were washed by floating on several drops of buffer and blocking solutions prior to routine labeling procedure ( Slot *et al.*, 1991) with primary antibodies (1:30) against HA (SC-805, Santa Cruz Biotechnology), and secondary anti-rabbit antibodies (1:30) coupled to 12 nm colloidal gold (111-205-144, Jackson ImmunoResearch, West Grove, PA, USA). After labeling, sections were washed on distilled water droplets, stained by immersing in a mixture of 1.8% methyl cellulose and 0.4% uranyl acetate ( Griffiths *et al.*, 1983) for 10 min, and dried for imaging by TEM (Philips, CM12). For controls, primary antibodies were omitted, and labeling was also performed in wtHSV-1 infected cells.

## Results

### CK177ΔUs3gK-HA induces alteration of nuclear and Golgi membranes

**Figure 1.**
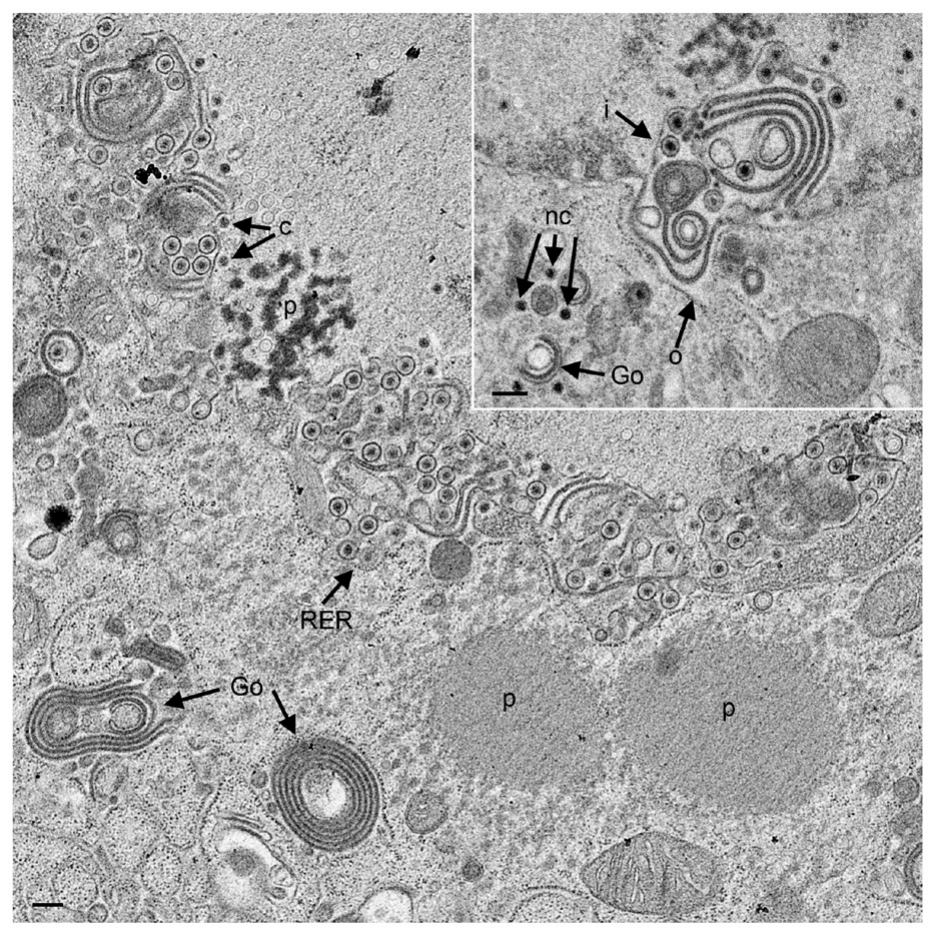
Transmission electron microscopy of Vero cells 24 hpi with CK177ΔUs3gK-HA. The inner nuclear membrane (INM) (i) formed invaginations and multiple folds shown in detail in the inset. Capsids (c) bud at the INM, and virions accumulate in the perinuclear space delineated by the INM and outer nuclear membrane (ONM) (o), and to a much less extent in adjacent cisternae of the endoplasmic reticulum (RER). A few naked capsids (nc) are in close vicinity to Golgi membranes (Go). Note the presence of electron dense material (p) with the appearance of proteins in the nucleus and the cytoplasm. Bars, 200 nm.

**Figure 2.**
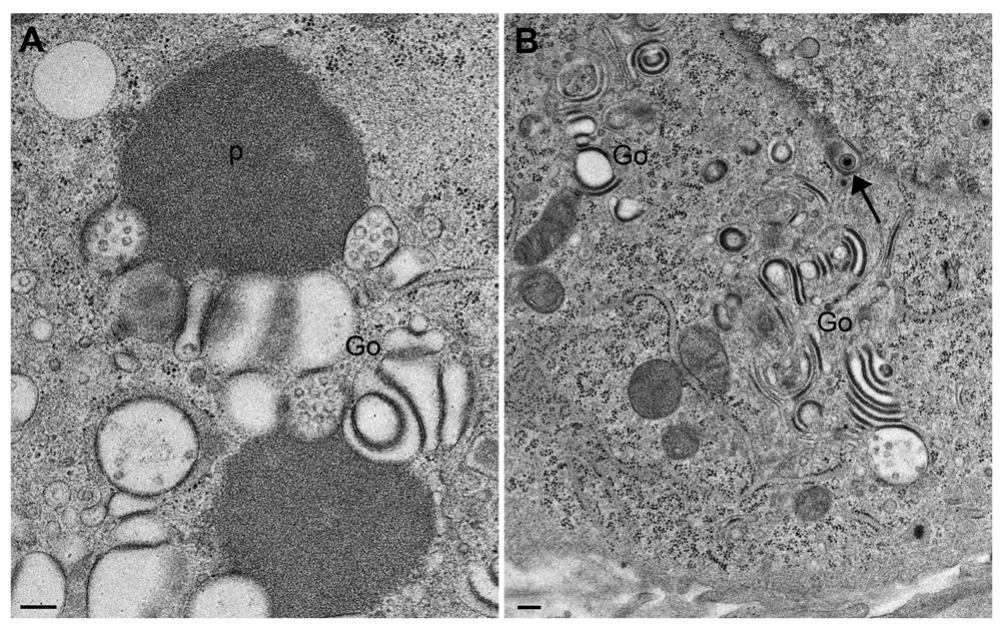
Transmission electron microscopy images of Vero cells 16 hpi with R7041ΔUs3. ( **A**) homogenous, proteinaceous deposits (p) adjacent to altered Golgi membranes (Go), and ( **B**) Golgi fields containing thick, electron dense membranes with the staining properties of proteins. Note the virion within double-coated structures (arrow), and the one in the endoplasmic reticulum (beside the arrowhead). Bars, 200 nm.

**Figure 3.**
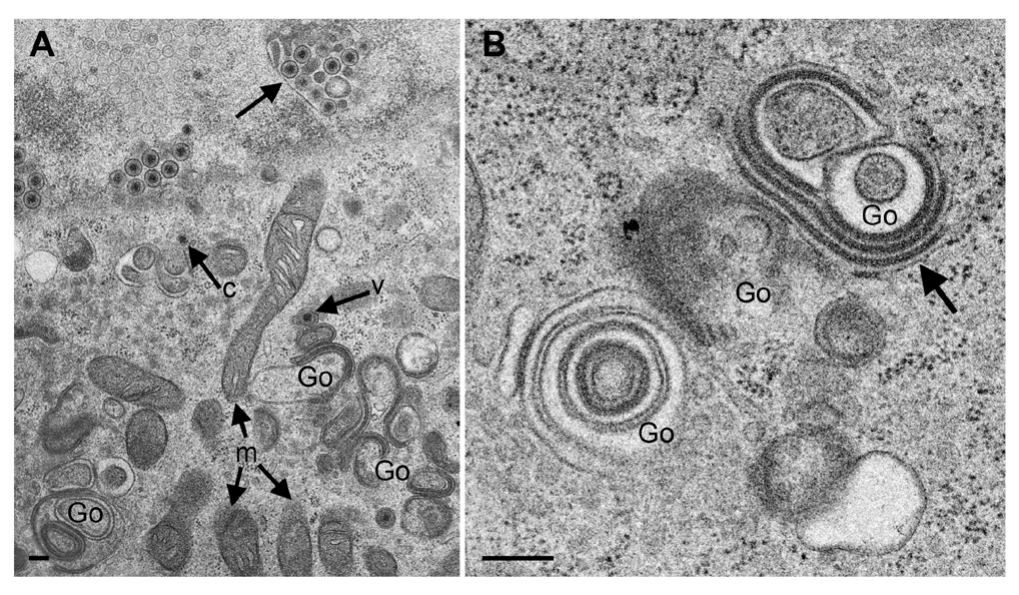
Transmission electron microscopy of Vero cells 24 hpi ( **A**) and 20 hpi ( **B**) with CK177ΔUs3gK-HA. The nucleus in ( **A**) is tangentially sectioned so that invaginations of the inner nuclear membrane (arrow) appear as hibernations. The Golgi complex (Go) comprises multiple stacks consisting of electron dense membranes similar as in R7041ΔUs3 infections. One capsid (c) is in the cytoplasm, and two virions (one indicated by “v”) are within small concentric vacuoles. Note the well-preserved mitochondria (m). ( **B**) shows details of Golgi membranes, which continue into rough endoplasmic reticulum membranes (thick arrow), and a tangentially sectioned Golgi field. Bars, 200 nm.

Infection with HSV-1ΔUs3 induces formation of folds and invagination of nuclear membranes associated with accumulation of virions ( Poon *et al.*, 2006; Reynolds *et al.*, 2002; Wisner *et al.*, 2009). Infection with CK177ΔUs3gK-HA, a similar mutant lacking Us3 but equipped with an epitope-tagged gK, also induced multiple folds and invaginations of nuclear membranes containing virions. Moreover, homogenous, proteinaceous structures occurred within nuclei and cytoplasm of CK177ΔUs3gK-HA ( Figure 1) and R7041ΔUs3 ( Figure 2A) infected cells. Golgi complexes consisted of multiple stacks comprising thick electron dense membranes especially at the trans face ( Figure 3) similarly as described in R7041ΔUs3 ( Figure 2B) infections ( Wild *et al.*, 2015). We conclude that the absence of Us3 is responsible for the alterations in the Golgi architecture, while the epitope-tagged gK-HA has no obvious impact on Golgi disorganization. Unprocessed images of this, and all other figures, are available as *Underlying data* ( Tobler *et al.*, 2019).

### gK localizes on Golgi membranes, nuclear membranes, virions, and proteinaceous deposits

**Figure 4.**
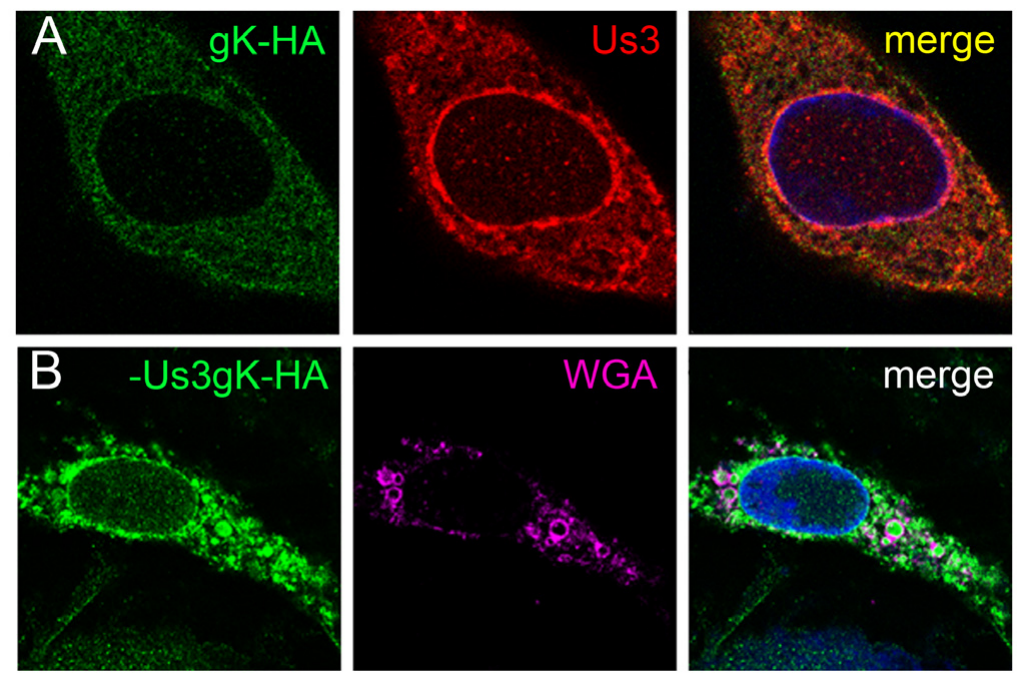
Immunofluorescence microscopy of Vero cell at 20 hpi with CK177gK-HA ( **A**) or CK177ΔUs3gK-HA ( **B**). CK177gK-HA infected cells show fine but dense distribution of gK-HA over the entire cytoplasm, at the nuclear rim and, at a less extent, in the nucleus. Distribution of Us3 is similar though more intense at the nuclear rim. In the absence of Us3, gK-HA signals are markedly enhanced in all three compartments. gK-HA partly localizes with wheat germ agglutinin (WGA) as a marker for Golgi membranes. The fate of the Golgi complex is under investigation using specific markers.

**Figure 5.**
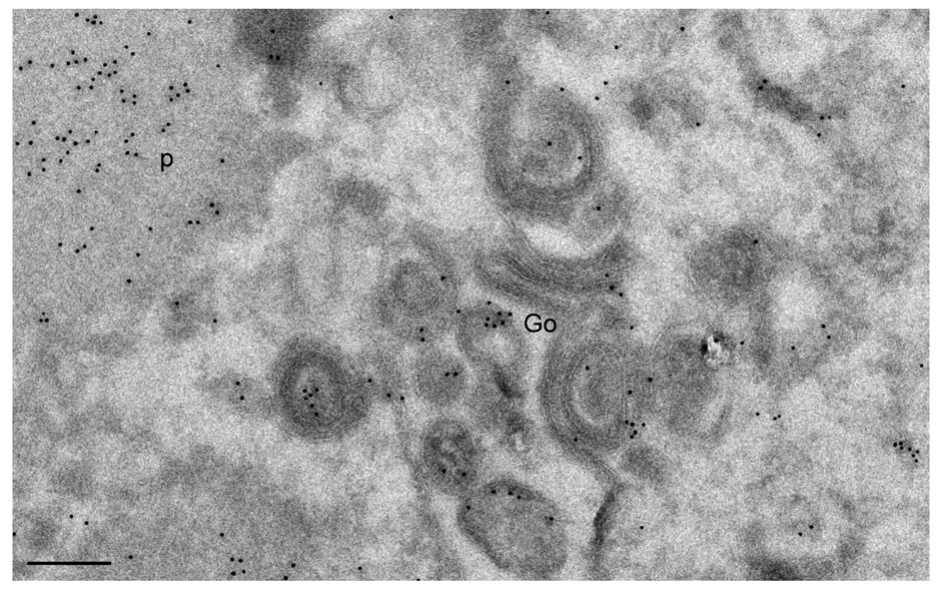
Golgi membranes 20 hpi with CK177ΔUs3gK-HA after immunogoldlabeling using antibodies against HA. Gold particles are irregularly distributed on electron dense Golgi membranes (Go) as well as on proteinaceous deposits (p). Bars, 200 nm.

**Figure 6.**
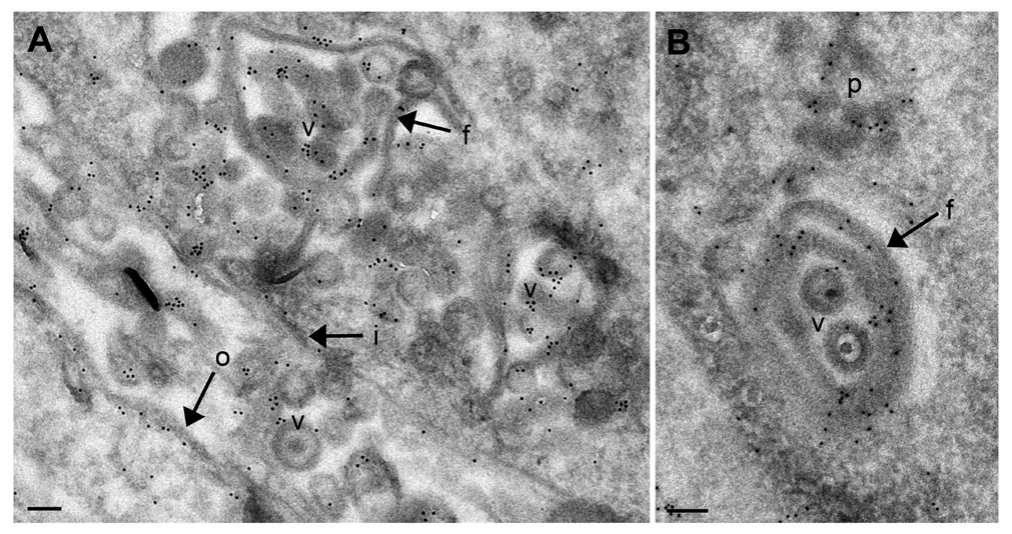
Transmission electron microscopy images of the nuclear periphery in Vero cells 20 hpi with CK177ΔUs3gK-HA after immunogoldlabeling using antibodies against HA. ( **A**) Shows an overview. ( **B**) Shows a detail of membrane folds protruding into the nucleus. Gold particles are located on nuclear membrane folds (f), virions (v), nuclear membranes (i, o) and on protein deposits (p) in the nucleus. Bars, 200 nm.

**Figure 7.**
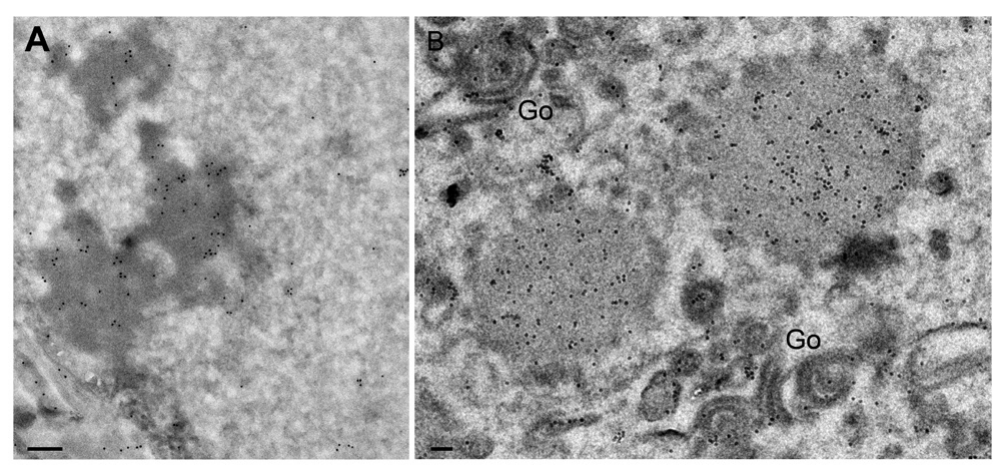
After infection with CK177ΔUs3gK-HA, homogenous structures are intensely labeled by antibodies against HA in nuclei ( **A**) and cytoplasm where they often neighboring Golgi membranes (Go). Bars, 200 nm.

Budding of capsids at nuclear and Golgi membranes starts by insertion of budding proteins that appears in electron micrographs as a dense layer ( Leuzinger *et al.*, 2005). The major budding proteins at nuclear membranes are UL31/UL34 ( Bigalke *et al.*, 2014; Hagen *et al.*, 2015), while gK and UL20 protein are responsible for budding at Golgi membranes ( Melancon *et al.*, 2007). Infection with CK177gK-HA revealed that gK localizes in cytoplasm, nuclear rim and even in nuclei ( Figure 4A) by immunofluorescence using antibodies against HA. Following infection with CK177ΔUs3gK-HA, gK-HA signals are strongly enhanced in the cytoplasm (forming large clusters), at the nuclear rim and in nuclei ( Figure 4B). At the ultrastructural level, Golgi membranes ( Figure 5), nuclear membranes and viral envelopes ( Figure 6) and the homogeneous structures in nuclei and cytoplasm ( Figure 7) were distinctly labeled in CK177ΔUs3gK-HA infected cells. Immunogold labeling agrees with the distribution of gK-HA visualized by immunofluorescence implying that gK was, in addition to Golgi localization, translocated into nuclei, incorporated into nuclear membranes, and became part of the viral envelope during budding.

## Discussion

The precise functions of Us3 kinase has been reviewed by Kato & Kawaguchi (2018). However, the mechanism of regulation phospholipid-biosynthesis is unknown ( Wild *et al.*, 2012). Membrane enlargement in the absence of Us3 is associated with structural alterations including folding of nuclear membranes and malformation of Golgi stacks, respectively, and insertion of proteins into nuclear and Golgi membranes. Immunolabeling using antibodies against the HA tag recognized gK to be one component of altered membranes suggesting that deposition/insertion of gK is related to membrane overproduction in the absence of Us3.

HSV-1ΔUs3 virions are infective even though they are not released from the PNS, and do not pass the Golgi complex. gK has been reported to be involved in viral envelopment by Golgi membranes, not by nuclear membranes ( Melancon *et al.*, 2005). gK is essential for infectivity playing a significant role in viral entry ( Foster *et al.*, 2001; Musarrat *et al.*, 2018). Therefore, gK must be provided during budding of HSV-1ΔUs3 at nuclear membranes. The intense labeling of gK on nuclear membranes and viral envelopes clearly demonstrates that gK becomes part of the viral envelope during budding of HSV-1ΔUs3. gK was also found in nuclei in both HSV-1ΔUs3 and CK177gK-HA infected cells indicating that gK is transported into the nucleus in the presence or absence of Us3. Indeed, others have also observed gK incorporated into nuclear membranes in the context of wtHSV-1 infection ( Rajcani & Kudelova, 1998) suggesting that gK may also play a significant role in envelopment of wtHSV-1 at nuclear membranes.

## Conclusion

Without Us3, Golgi and nuclear membranes proliferate in association with incorporation of gK that also localizes on virions remaining stuck in the PNS but mature to infectivity suggesting that i) Us3 kinase is involved in regulation in nuclear and Golgi membranes assembly possibly in association with gK synthesis and/or distribution, ii) gK may be the target of Us3 phosphorylation, which is needed for virions to proceed out of the PNS, and iii) gK may also be involved in nucleus-to-cytoplasm translocation in wtHS-1 because gK localizes on nuclei and nuclear rim in the presence of Us3. We hypothesize that Us3 interplays with mechanisms regulating synthesis and arrangement of Golgi membranes, nuclear membranes and gK, which might be the most significant role of Us3 remaining to be investigated.

## Data availability

Figshare: The herpes simplex virus 1 Us3 kinase is involved in assembly of membranes needed for viral envelopment and in distribution of glycoprotein K. https://doi.org/10.6084/m9.figshare.8131241.v2 ( Tobler *et al.*, 2019).

This project contains the raw images captured for each Figure, with the number of the Figure indicated on each file name.

Data are available under the terms of the Creative Commons Attribution 4.0 International license (CC-BY 4.0).
