# Supplementary material for: The herpes simplex virus 1 Us3 kinase is involved in assembly of membranes needed for viral envelopment and in distribution of glycoprotein K
Source: F1000Res. 2019 May 23;8:727. [Version 1] doi: 10.12688/f1000research.19194.1 (PMC6681629; doi:10.12688/f1000research.19194.1)
Supplement: Peer review history

# Peer review: Reviewer response for version 1

**DOI:** 10.5256/f1000research.21034.r51383

**Published:** 2019-08-02

**Authors:** Benedikt Kaufer

**Affiliations:** 1 Institute of Virology, Department of Veterinary Medicine, Free University of Berlin, Berlin, Germany, Berlin, Germany

In this manuscript, the authors generated two herpes simplex virus 1 (HSV-1) mutants harboring a deletion of US3 and insertion of a fluorescent tag on gK. They used these viruses to assess the effect of the US3 deletion on the gK expression levels and localization. Intriguingly, they observed an increased expression of gK and localization of the protein to the nuclear lamina. The manuscript is exciting and well written. However, as the other reviewers pointed out, it would be worth sequencing the recombinant BACs (or the resulting viruses) to ensure that the effect is not due to secondary mutations in the virus genome. Nowadays this would be quick and relatively cheap to sequence the virus genome by miSeq and would eliminate the need for a revertant virus.

I confirm that I have read this submission and believe that I have an appropriate level of expertise to confirm that it is of an acceptable scientific standard, however I have significant reservations, as outlined above.

# Peer review: Reviewer response for version 1

**DOI:** 10.5256/f1000research.21034.r49006

**Published:** 2019-07-08

**Authors:** Antonio Alcamí

**Affiliations:** 1 Centre for Molecular Biology, Spanish Scientific Research Council (CSIC)-Autonomous University of Madrid (UAM)), Madrid, Spain

HSV-1 accumulates in the perinuclear space when US3 is deleted. The authors investigate the distribution of gK in the context of US3 deletion. gK is a glycoprotein proposed to be involved in the envelopment of capsids by Golgi membranes, but not by nuclear membranes. The incorporation of gK into the virus envelope in spite of virus morphogenesis being arrested in the perinuclear space in the absence of US3 suggests new functions of gK during budding of HSV-1 at the nuclear membranes. This is a novel observation in relation to the function of gK in viral morphogenesis.

 The authors generated two new recombinant viruses, CK177deltaUS3gK-HA and CK177gK-HA, using the BAC system. A major concern of the experimental work presented is the limited characterization of the genomic structure of these recombinant viruses. The possibility that undesired mutations in other loci of the viral genome may have been selected during the construction of the recombinant viruses should be controlled by either sequencing of the viral genome or the construction of a revertant virus in which the modification (introduction of gK-HA with or without the deletion of US3) is restored, and demonstration that the revertant viruses behave as WT viruses. Also, there is no evidence that US3 protein expression is absent in CK177deltaUS3gK-HA. A detailed characterization of the recombinant viruses is necessary to fully support the conclusions of the study, and to rule out the introduction of mutations in other genes.

 The presentation of more quantitative data to support the distribution of gK by immunoelectron microscopy would strengthen the conclusions.

I confirm that I have read this submission and believe that I have an appropriate level of expertise to confirm that it is of an acceptable scientific standard, however I have significant reservations, as outlined above.

# Peer review: Reviewer response for version 1

**DOI:** 10.5256/f1000research.21034.r49387

**Published:** 2019-07-08

**Authors:** Konstantin G. Kousoulas

**Affiliations:** 1 School of Veterinary Medicine, Department of Pathobiological Sciences, Louisiana State University, Baton Rouge, LA, USA

In this manuscript results are presented indicating that deletion of the HSV-1 US3 causes internal membrane proliferation and rearrangements and is accompanied by over expression of glycoprotein K (gK). The results point into a potential functional relationship between US3 and gK that has not been previously reported. Authors should note that a previous publication showed that overproduction of gK causes accumulation of enveloped capsids inside the nuclear membranes.

 This reference should be included and discussed. Authors should also sequence the gK region to ensure that the overproduction of gK is not independent of US3 due to secondary mutations in the genomic area.

I confirm that I have read this submission and believe that I have an appropriate level of expertise to confirm that it is of an acceptable scientific standard.

## References

[1] : Overexpression of gK in gK-transformed cells collapses the Golgi apparatus into the endoplasmic reticulum inhibiting virion egress, glycoprotein transport, and virus-induced cell fusion. *Virology*.2003;317(2) :237-52 1469866310.1016/j.virol.2003.07.008
